# Common risk alleles for schizophrenia within the major histocompatibility complex predict white matter microstructure

**DOI:** 10.1038/s41398-024-02910-2

**Published:** 2024-04-22

**Authors:** Xavier Caseras, Emily Simmonds, Antonio F. Pardiñas, Richard Anney, Sophie E. Legge, James T. R. Walters, Neil A. Harrison, Michael C. O’Donovan, Valentina Escott-Price

**Affiliations:** 1 Centre for Neuropsychiatric Genetics and Genomics, Department of Psychological Medicine and Clinical Neurosciences, Cardiff University, Cardiff, UK; 2 Dementia Research Institute, London, UK; 3 Cardiff University Brain Research Imaging Centre, Cardiff University, Cardiff, UK

**Keywords:** Schizophrenia, Predictive markers

## Abstract

Recent research has highlighted the role of complement genes in shaping the microstructure of the brain during early development, and in contributing to common allele risk for Schizophrenia. We hypothesised that common risk variants for schizophrenia within complement genes will associate with structural changes in white matter microstructure within tracts innervating the frontal lobe. Results showed that risk alleles within the complement gene set, but also intergenic alleles, significantly predict axonal density in white matter tracts connecting frontal cortex with parietal, temporal and occipital cortices. Specifically, risk alleles within the Major Histocompatibility Complex region in chromosome 6 appeared to drive these associations. No significant associations were found for the orientation dispersion index. These results suggest that changes in axonal packing - but not in axonal coherence - determined by common risk alleles within the MHC genomic region **–** including variants related to the Complement system - appear as a potential neurobiological mechanism for schizophrenia.

## Introduction

Schizophrenia is a highly polygenic heritable disorder, with a heritability estimated around 80% by twin studies [1]. The most recent genome-wide association study (GWAS) from the Psychiatric Genetics Consortium [2] identified just over 300 significant independent SNPs associated with schizophrenia, enabling the calculation of polygenic risk scores (PRS; i.e. an estimate of an individual’s cumulative common genetic liability [3]) that robustly associate with brain phenotypes in population samples, endorsing the impact of common allele risk for schizophrenia on brain’s neuroanatomy [4].

Little mechanistic interpretation of the above association between schizophrenia PRS and neuroanatomy is possible, though, given that the genome-wide approach to the PRS calculation reflects the effects of genetic variation on hundreds or thousands of genes involved in many different biological processes. We have previously argued that focusing on gene-set PRS would allow a richer mechanistic interpretation of the results based on the ontology of the genes included in the PRS, as well as preventing a potential dilution of the associations due to the inclusion in the same score of associated risk variants along many other variants with null effects, or even with opposite direction of effect, over the same brain phenotype [5].

Recent research has shown the complement system to play a key role in neuronal proliferation, migration and pruning during early brain development [6, 7], and variation within complement genes (the most compelling evidence pointing at C4) to be associated with increased risk for schizophrenia [8, 9], with a putative mechanism being overactivation of this system resulting in excessive synaptic pruning [10]. Schizophrenia has long been conceptualised as a disorder of brain dysconnectivity, notably affecting the connectivity of the frontal lobe [11, 12]. Recent diffusion tensor imaging research has shown widespread differences across white matter tracts in people with schizophrenia compared with control participants [13]. We postulate these to be linked observations, that is, perturbed complement function is causally related to white matter microstructural abnormalities in schizophrenia.

In this study we aimed to investigate whether common variant risk for schizophrenia within complement genes is associated with microstructural changes as indexed by axonal packing and coherence in association white matter tracts innervating the frontal cortex. We hypothesised that the previously reported association between schizophrenia genome-wide PRS and axonal density [4] will be mostly driven by genic signals as opposed to intergenic signals, and that within those, common alleles within complement genes will be the main drives of the association. We do not predict any associations with orientation dispersion.

## Methods

### Sample

Participants were drawn from the UK Biobank cohort. Participants were excluded if self-reported to had received a diagnosis, or if clinical records were available, of substance dependency (including alcohol), psychosis (including bipolar disorder), learning disability or neurodegenerative conditions. Only unaffected participants of British and Irish genetic ancestry, unrelated up to 2nd degree relatives and for whom Neural Orientation and Dispersion Density Imaging (NODDI) [14] data were available at the time of analyses, were retained for the study (*n* = 32,637).

All participants provided informed consent to participate in UK Biobank. Ethical approval was granted to the UK Biobank project by the North West Multi-Centre Ethics committee. Data were released to us after application project reference 17044.

### Schizophrenia polygenic scores

Genotyping was performed by UK Biobank using Affymetrix UK Axiom arrays. Further to UK Biobank’s processing [15] we excluded SNPs with MAF < 5% and HWE *p*-value < 10^−^^6^. Correlated SNPs were clumped using r^2^ ≤ 0.1 and a physical distance of 1 Mb, retaining those SNPs most associated with the disorder based on corresponding summary statistics for the latest schizophrenia GWAS from the Psychiatric Genetics Consortium (PGC3) [2] after excluding participants also included in UK Biobank. PRS were calculated on Plink2.0 using consecutive p-value thresholds of 1 × 10^−^^5^, 1 × 10^−^^4^, 1 × 10^−^^3^, 0.01, and 0.1 and weighted with the effect sizes from the GWAS summary statistics mentioned above. PRS were adjusted for the first 15 principal components and genotype array, and standardised.

Four PRS were initially calculated: Genome-wide, Genic, Intergenic and Complement. The Genome-wide PRS included any SNPs with an associated p-value equal or smaller to the p-threshold applied, as conventionally defined in PRS research studies. This was then split into its genic and intergenic compartments. In the Genic PRS we restricted SNP selection to those located within 35 kb upstream and 10 kb downstream from genes detailed in the GENCODEv31 human gene set [16] (this score also included SNPs within the Complement genes). The same procedure was applied to the Complement PRS but restricted to genic SNPs within a comprehensive list of Complement genes [17] (Supplemental Table 1). SNPs outside the Genic boundaries were used to calculate the Intergenic PRS. Clumping of SNPs was always performed within each set (i.e., Genome-wide, Genic, Complement, Intergenic). Due to the complex linkage disequilibrium structure within the Major Histocompatibility Complex (MHC) region, we additionally computed all the above PRS after excluding this section of the genome (chr6:25-35 Mb). The number of SNPs included in each PRS is presented in supplemental Table 2.

### Brain phenotypes

Derived measures available from UK Biobank of weighted-mean for intracellular volume fraction and orientation dispersion obtained via Neurite Orientation Dispersion and Density Imaging (NODDI) [14] were used. Intracellular volume fraction is proposed to index axonal density in white matter (i.e. packing and myelination of fibers contained within white matter tracts), whereas orientation dispersion quantifies the bending and fanning of axons and therefore can be considered an index of axonal coherence within white matter; both these metrics being components of the more traditional measure of fractional anisotropy, allowing a finer-grain approach to white mater micro-structure. To avoid the effects of outliers potentially resulting from noisy data or deficient tractography, values exceeding ±3 SD from the group mean in any of these measures were excluded from the analyses. The averaged left and right hemispheres axonal density and orientation dispersion were finally calculated for the main association tracts innervating the frontal cortex: cingulum-cingulate gyrus part (CG), inferior fronto-occipital fasciculus (iFO), superior longitudinal fasciculus (sL) and uncinate fasciculus (Unc).

### Analyses

Separate linear regression analyses with brain phenotypes as outcome and PRS as main predictor were run for each white matter tract. We included sex, age and scan site as covariates into each regression model. We report the β values and their associated two-tailed p-value as effect size indices of the association between the standardised outcomes and predictors. Despite the PRS calculated at different threshold levels are not independent from each other, we chose to apply a stringent approach to multiple testing (i.e., Bonferroni correction) within each hypothesis tested in this study.

As a complementary approach to evaluate the robustness of our main PRS-based results, we analysed local patterns of genomic correlation between schizophrenia and axonal density using LAVA v0.1.0 [18] on the R statistical computing environment v4.3.0. Axonal density GWAS were ran on the same UK Biobank sample used for PRS association analyses (Supplementary Methods). Summary statistics from these GWAS were processed by LAVA together with Schizophrenia summary statistics from PGC3 [2], leaving 3.8 M overlapping SNPs for analysis. The LAVA algorithm was then ran as described in its parent publication [18]. Briefly, the local heritability of all phenotypes was first evaluated on a list of 2495 quasi-independent loci, generated by partitioning the genome in ~1 Mb blocks with minimal between-block LD. Loci in which the local heritability of both schizophrenia and at least a brain phenotype survived a Bonferroni-corrected p-value threshold of *p* < 2.004 × 10^−^^4^ (0.05/2495) were then evaluated for pairwise local genomic correlations (r_g_). Statistical significance for the computed local r_g_ was also adjusted via the Bonferroni correction. As the MHC region is represented in this list of loci by 22 LD blocks, we also performed separate univariate and bivariate tests for all phenotype combinations with it defined as a single block.

## Results

As starting point we tested the replication of previously published results showing the association of genome-wide PRS for schizophrenia with axonal density. As expected, we found significant negative associations between this PRS and axonal density across most p-thresholds in all tracts, except for Unc. However, only in the case of sL some of the associations surpassed the Bonferroni correction threshold (Table 1). No consistent associations were found for orientation dispersion except for a moderate increased orientation dispersion in CG (Table 1).

**Table 1:** Genome-wide polygenic risk scores for schizophrenia calculated at 5 progressive p thresholds, and their association (beta[p]) with axonal density in association tracts.

	CG	iFO	sL	Unc
**Axonal density**				
SZ PRS				
0.1	−0.008 (0.13)	−0.009 (0.08)	−0.014 (0.008)	0.001 (0.85)
0.01	−0.012 (0.03)	−0.015 (0.006)	**−0.018 (0.0007)**	−0.006 (0.28)
0.001	−0.010 (0.05)	−0.013 (0.01)	−0.015 (0.006)	−0.006 (0.26)
0.0001	−0.016 (0.003)	−0.012 (0.02)	−0.016 (0.004)	−0.006 (0.23)
0.00001	−0.014 (0.01)	−0.016 (0.003)	**−0.017 (0.002)**	−0.008 (0.11)
**Orientation dispersion**				
SZ PRS				
0.1	0.006 (0.26)	0.009 (0.1)	−0.002 (0.66)	0.010 (0.06)
0.01	0.014 (0.01)	0.009 (0.10)	−0.007 (0.21)	0.008 (0.12)
0.001	0.014 (0.01)	0.009 (0.08)	−0.001 (0.88)	0.008 (0.14)
0.0001	0.016 (0.004)	0.009 (0.08)	−0.006 (0.29)	0.013 (0.01)
0.00001	0.014 (0.01)	0.007 (0.22)	0.003 (0.61)	0.016 (0.004)

*CG* Cingulum-cingulate gyrus part, *iFO* Inferior fronto-occipital fasciculus, *sL* Superior longitudinal fasciculus, *Unc* Uncinate fasciculus.

Bonferroni corrected (20 tests) significant associations highlighted in bold.

**Fig. 1:**
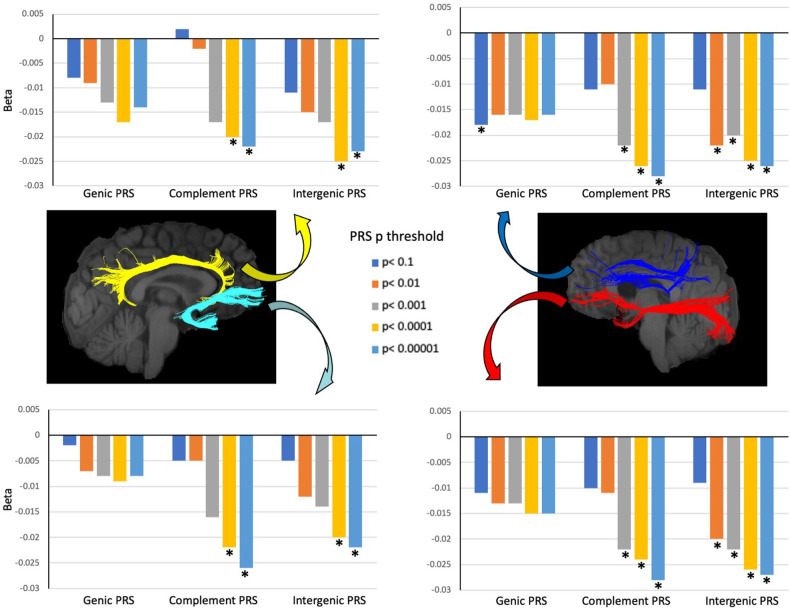
Association (Beta) between polygenic risk scores for schizophrenia (genic, complement and intergenic) and axonal density in cingulum-cingulate gyrus part (yellow), inferior fronto-occipital fasciculus (red), superior longitudinal fasciculus (dark blue) and uncinate fasciculus (light blue). The asterisk indicates significant Beta values at a Bonferroni corrected threshold of 0.00083. White matter tracts in this figure have been reproduced from a single participant using ExploreDTI (http://exploredti.com) for visual illustration purposes only.

To test our main hypothesis, we investigated the associations of genic, intergenic and complement PRS with axonal density. The genic and intergenic PRS were negatively associated with axonal density in all tracts analysed. All but one associatio**n** involving genic PRS felt short of the Bonferroni significant threshold, whereas intergenic PRS showed strong associations with all tracts, with over half of the tests remaining significant after Bonferroni correction. Restricting the genic PRS to variants within the complement gene-set resulted in generally larger effect sizes across all tracts with half of the tests remaining significant after Bonferroni correction (Fig. 1 & Supplementary Table 3). To examine whether complement and intergenic PRS were independent predictors of axonal density from each other and genic PRS, we ran regression analyses simultaneously entering these three PRS into the model. Results confirmed that complement and intergenic PRS were predictive of axonal density independently of each other, both predicting axonal density’s variability beyond that explained by genic PRS (Supplementary Table 4).

On inspection of the list of signals included in the above intergenic and complement PRS ordered by their contribution to the total score, SNPs within the extended MHC region of chromosome 6 showed to accumulate among the top signals (Supplemental Table 5). Due to the complex linkage disequilibrium structure within this region, we recalculated the genic, intergenic and complement PRS after excluding the MHC region. The associations of the complement-MHC PRS with axonal density were considerably attenuated, and none were significant after Bonferroni correction. The associations of intergenic-MHC PRS were also attenuated to a degree, but nevertheless remained a significant predictor of axonal density (Table 2).

**Table 2:** Genic, Complement and Intergenic schizophrenia polygenic risk scores after removing the MHC regions (chr6:25-35 Mb) calculated at 5 progressive p thresholds, and their association (beta[p]) with axonal density in association tracts.

	CG	iFO	sL	Unc
**Genic PRS**				
0.1	−0.005 (0.39)	−0.007 (0.18)	−0.015 (0.006)	0.001 (0.79)
0.01	−0.005 (0.37)	−0.008 (0.13)	−0.012 (0.03)	−0.003 (0.61)
0.001	−0.008 (0.15)	−0.007 (0.21)	−0.011 (0.05)	−0.003 (0.57)
0.0001	−0.012 (0.02)	−0.007 (0.17)	−0.011 (0.04)	−0.003 (0.54)
0.00001	−0.010 (0.07)	−0.008 (0.12)	−0.010 (.07)	−0.003 (0.63)
**Complement PRS**				
0.1	0.005 (0.38)	−0.002 (0.68)	<0.001 (0.95)	0.001 (0.92)
0.01	0.006 (0.26)	−0.001 (0.89)	<0.001 (0.93)	0.003 (0.53)
0.001	−0.009 (0.14)	−0.010 (0.05)	−0.010 (.05)	−0.008 (.13)
0.0001	−0.010 (0.07)	−0.008 (0.14)	−0.011 (.04)	−0.011 (.04)
0.00001	−0.008 (0.11)	−0.007 (0.19)	−0.010 (.07)	−0.012 (.02)
**Intergenic PRS**				
0.1	−0.008 (0.13)	−0.007 (0.21)	−0.008 (0.15)	−0.001 (0.82)
0.01	−0.011 (0.03)	−0.016 (.002)	−0.018 (0.001)	−0.008 (.12)
0.001	−0.012 (0.02)	−0.016 (.002)	−0.015 (0.007)	0.009 (0.09)
0.0001	**−0.019 (0.0003)**	**−0.020 (0.0002)**	**−0.020 (0.0003)**	−0.016 (0.003)
0.00001	−0.017 (0.002)	**−0.020 (0.0002)**	**−0.019 (0.0004)**	−0.016 (0.003)

*CG* Cingulum-cingulate gyrus part, *iFO* Inferior fronto-occipital fasciculus, *sL* Superior longitudinal fasciculus, *Unc* Uncinate fasciculus.

Bonferroni corrected (60 tests) significant results are highlighted in bold.

To further examine the role of the MHC region in axonal density, we calculated a schizophrenia PRS-like score based on the four SNPs within the extended MHC region that had been originally reported by the PGC3 [2] to have conditionally independent evidence for association with SZ (i.e., rs13195636, rs8192589, rs9461856, rs9368789). This PRS-like score was strongly associated with axonal density (Table 3). Individually, the risk allele count for rs13195636 and rs8192589 were also significantly associated with axonal density in all association tracts (Table 3), but not the risk allele count of the other two SNPs. A regression analysis including the risk allele counts for both rs13195636 and rs8192589 into the same regression model showed both SNPs to independently predict reduced axonal density in most tracts (Supplemental table 6).

**Table 3:** Association (beta[p]) with axonal density in association tracts of four quasi-independent risk SNP for schizophrenia within the MHC region and of their added risk.

	CG	iFO	sL	Unc
Added risk	**−0.019 (0.0003)**	−0.028 (2*10^**−6**^**)**	−0.027 (7*10^**−7**^**)**	−0.023 (1*10^**−5**^**)**
rs13195636	−0.026 (9*10^**−7**^**)**	−0.030 (2*10^**−8**^**)**	−0.026 (1*10^**−6**^**)**	−0.027 (3*10^**−7**^**)**
rs8192589	**−0.018 (0.0008)**	−0.030 (2*10^**−8**^**)**	−0.029 (1*10^**−7**^**)**	−0.023 (1*10^**−5**^**)**
rs9461856	−0.009 (0.07)	−0.012 (0.02)	−0.001 (0.84)	−0.013 (0.01)
rs9368789	0.013 (0.01)	0.009 (0.10)	0.013 (0.01)	0.007 (0.22)

Risk variant rs13195636 (A), rs8192589 (G), rs9461856 (G), rs9368789 (C).

*CG* Cingulum-cingulate gyrus part, *iFO* Inferior fronto-occipital fasciculus, *sL* Superior longitudinal fasciculus, *Unc* Uncinate fasciculus.

Bonferroni corrected (20 tests) significant results are highlighted in bold.

To examine the association of schizophrenia risk within complement genes located in the MHC region with axonal density, and since none of the four SNPs highlighted above is located within them, we calculated the schizophrenia gene-based PRS for C2, C4A, C4B and CFB separately and added together into a single risk score using the same method as before. The added risk of those four genes, as well as all individual gene PRS but that of C4A showed strong negative association with axonal density in all tracts analysed (Table 4). A further regression model in which all four PRS were included to account for the interrelationship among them, resulted in C2 maintaining the strongest associations with axonal density in all tracts, albeit only the association with CG axonal density survived Bonferroni correction (Supplemental tables 7 & 8).

**Table 4:** Association (beta[p]) of schizophrenia polygenic risk score for each Complement gene located in the MHC region separately with axonal density in association tracts.

	n SNPs	CG	iFO	sL	Unc
Added risk		**−0.019 (0.0003)**	−0.028 (2*10^**−7**^**)**	−0.027 (7*10^**−7**^**)**	−0.023 (1*10^**−5**^**)**
C2	2	−0.024 (1*10^**−5**^**)**	−0.033 (5*10^**−10**^**)**	−0.030 (2*10^**−8**^**)**	−0.025 (3*10^**−6**^**)**
C4A	1	−0.001 (0.92)	−0.006 (0.24)	−0.002 (0.70)	−0.005 (0.36)
C4B	3	−0.022 (4*10^**−5**^**)**	−0.032 (2*10^**−9**^**)**	−0.030 (2*10^**−8**^**)**	−0.024 (8*10^**−6**^**)**
CFB	2	**−0.020 (0.0001)**	−0.031 (4*10^**−9**^**)**	−0.028 (1*10^**−7**^**)**	−0.024 (1*10^**−5**^**)**

*n SNPs* number of SNPs included in the PRS after clumping.

*CG* Cingulum-cingulate gyrus part, *iFO* Inferior fronto-occipital fasciculus, *sL* Superior longitudinal fasciculus, *Unc* Uncinate fasciculus.

Bonferroni corrected (**20 tests**) significant results are highlighted in bold.

Due to the unexpected significant associations obtained with intergenic PRS and to further explore whether the intergenic PRS–MHC association with axonal density was a diffuse or regionally concentrated, we calculated an intergenic PRS at its most predictive p-threshold (< 0.0001) for each autosome separately (in the case of chromosome 6 after excluding the MHC region). Intergenic SNPs within chromosome 19 predicted axonal density across all tracts, whereas intergenic PRS for chromosome 4 associated with axonal density in iFO and sL, and for chromosome 1 with GC and Unc. All the intergenic signal from chromosome 6 was explained by SNPs within the MHC region (Table 5). To ascertain whether the intergenic associations found were independent of genic signals, we calculated genic PRS for chromosomes 1, 4 and 19 and ran the regression analyses above adding both intergenic and genic PRS as predictors in the same model. For chromosome 1, most the variance in axonal density was explained by genic PRS, and the previous associations with intergenic PRS were no longer significant. For chromosomes 4 and 19, the intergenic PRS associations remained significant independently of genic PRS, that showed weak associations (Supplemental table 9).

**Table 5:** Associations (beta[p]) between autosome intergenic polygenic risk scores for schizophrenia calculated at p threshold <0.0001 and axonal density in association tracts.

	CG	iFO	sL	Unc
**Chromosome**				
1	**−0.17 (0.001)**	−0.011 (0.03)	−0.013 (0.01)	**−0.017 (0.002)**
2	−0.003 (0.52)	0.001 (0.92)	−0.005 (0.39)	0.003 (0.61)
3	−0.006 (0.28)	−0.005 (0.39)	−0.008 (0.14)	−0.004 (0.39)
4	−0.007 (0.21)	**−0.014 (0.009)**	**−0.017 (0.002)**	−0.008 (0.12)
5	0.002 (0.73)	−0.006 (0.24)	−0.005 (0.39)	−0.001 (0.88)
6	**−0.025 (0.000003)**^**$**^	−0.029 (4*10^**−8**^**)**^**$**^	**−0.024 (0.000009)**^**$**^	−0.027 (5*10^**−7**^**)**^**$**^
6 - MHC	−0.007 (0.19)	−0.005 (0.34)	−0.002 (0.76)	−0.010 (0.05)
7	−0.010 (0.07)	−0.004 (0.47)	−0.004 (0.51)	−0.006 (0.27)
8	0.004 (0.41)	0.008 (0.13)	0.008 (0.13)	0.009 (0.09)
9	−0.010 (0.06)	−0.009 (0.10)	−0.014 (0.01)	−0.006 (0.22)
10	0.001 (0.83)	0.006 (0.28)	0.005 (0.34)	0.004 (0.47)
11	−0.007 (0.22)	−0.002 (0.67)	−0.005 (0.39)	−0.004 (0.48)
12	−0.008 (0.16)	−0.009 (0.08)	−0.008 (0.14)	−0.008 (0.15)
13	−0.001 (0.91)	0.006 (0.23)	0.003 (0.60)	0.004 (0.47)
14	0.002 (0.76)	−0.005 (0.32)	−0.003 (0.57)	0.001 (0.87)
15	−0.002 (0.66)	−0.008 (0.15)	−0.005 (0.39)	−0.003 (0.56)
16	0.006 (0.24)	0.002 (0.73)	0.002 (0.70)	−0.001 (0.79)
17	−0.002 (0.72)	0.003 (0.63)	< 0.001 (0.96)	0.001 (0.84)
18	−0.005 (0.38)	0.001 (0.92)	−0.002 (0.70)	−0.001 (0.83)
19	**−0.015 (0.006)**	**−0.019 (0.0004)**	**−0.018 (0.001)**	**−0.018 (0.0009)**
20	−0.003 (0.63)	−0.004 (0.41)	.001 (0.80)	0.001 (0.83)
21	0.002 (0.75)	−0.022 (0.75)	−0.001 (0.78)	0.002 (0.70)
22	−0.005 (0.33)	−0.003 (0.57)	−0.001 (0.91)	−0.003 (0.58)

*6-MHC* Chomosome 6 after excluding the MHC region, *CG* Cingulum-cingulate gyrus part, *iFO* Inferior fronto-occipital fasciculus, *sL* Superior longitudinal fasciculus, *Unc* Uncinate fasciculus.

Associations with *p* < 0.01are highlighted in bold for easier identification; ^$^Bonferroni corrected (88 tests) significant association.

As predicted based on previous results and our hypotheses, we did not find any robust associations across p-value thresholds between genic, complement or intergenic PRS for schizophrenia and orientation dispersion within any of the white matter tracts examined (Supplementary Table 10).

Local genomic correlation results from LAVA confirmed the relevance of the MHC region in carrying the association between common risk for schizophrenia and axonal density. Eight of the 22 LD blocks across this region showed significant bivariate associations between schizophrenia and all phenotypes, with local genetic correlations ranging between r_g_ = −0.523 and r_g_ = −1. Additional significant associations were seen in regions from chromosomes 4, 5, 10, 17 and 22 (Supplementary table 11). Assessing the MHC as a single LD block also yielded significant bivariate estimates for all phenotype comparisons (CG: r_g_ = −0.348 [−0.623, −0.116] *p* = 0.005; iFO: r_g_ = −0.520 [−0.745, −0.325] *p* = 7.35 × 10^−^^7^; sL: r_g_ = −0.406 [−0.616, −0.206] *p* = 7.26 × 10^−^^5^; Unc: r_g_ = −0.468 [−0.825, −0.214] *p* = 4.58 × 10^−^^4^).

## Discussion

In this study we compared the performance of a genome-wide PRS for schizophrenia against PRS restricted to genic, intergenic and complement gene-set variants in predicting axonal density and orientation dispersion in white matter association tracts innervating the frontal cortex. We show the complement gene-set PRS and the intergenic PRS to be the best predictors of axonal density, while no strong associations between any PRS and orientation dispersion were found. We further showed most of the complement signal to be driven by SNPs within the extended MHC region in chromosome 6. Significant local genomic correlation between schizophrenia and all axonal density phenotypes, potentially indicative of pleiotropic genetic variation, was also found in this region by directly analysing GWAS results using the LAVA method.

Several previous attempts to investigate the association between liability to schizophrenia indexed by PRS and neuroanatomical brain markers have failed to produce any robust results [19–22]. More recently, though, Stauffer et al. [4] reported robust associations by combining the increased power of the PGC3 [2] schizophrenia GWAS with the use of fine-grain measures of microstructure such is neurite density. We have previously proposed that restricting polygenic risk scores to gene-sets with a putative relation to the brain marker of interest - as opposed to the genome-wide approach - would enhance the discovery and the mechanistic interpretation of the association between common risk and brain phenotypes [5]. Here we develop this idea further showing that restricted PRS as opposed to the genome-wide PRS, clearly boosted the associations’ effect size with axonal density in a-priori selected white matter tracts. Despite further research is needed involving different brain phenotypes and gene-sets before conclusions are drawn, previous results from our group [5] along with the ones reported here support our approach to restrict PRS calculations to homogeneous groups of SNPs to maximise common variant risk associations with brain phenotypes.

Recent research has underlined the role of the complement system on neural development [6, 7], suggesting that an overactivation may result in over-pruning of neural connexions [10] during adolescence, that could explain the changes in functional connectivity typically observed in schizophrenia [23] and ultimately contribute to the presence of this disorder [8]. Expression of the *C4A* gene has been placed at the centre of this association [8, 24], based on its potential role in targeting synapses for microglial engulfment. However, *C4A* is not the only complement component contributing to this process [10]. Previous research has also shown other complement genes to associate with neuroanatomical changes (eg. CR1 with grey matter volume in the entorhinal cortex, and SERPING1 with cortical thickness in superior frontal cortex) [25, 26]. Our results showed that complement PRS was a strong predictor of axonal density, supporting the role of common risk variants for schizophrenia in shaping the microstructure of white matter tracts affecting frontal cortex’s connectivity. Further analyses showed this signal to be driven by SNPs within the MHC region, with no signal remaining significant outside this region. The complex linkage disequilibrium structure within this region challenges our ability to determine whether this association is driven by SNPs within complement genes, SNPs outside these genes, or a combination of both. We probed SNPs within this region that have conditionally independent evidence for association with schizophrenia, and identified two as strong independent predictors of axonal density (i.e. rs13195636 and rs8192589). None of these, though, are located within complement genes. The former is an intergenic variant closest to *ZNF184* and the latter is an intronic variant mapped to *NOTCH4*. Interestingly, both these genes have recently been associated with levels of complement proteins in serum [27, 28], and therefore it is possible that their mechanism of association with axonal density is via the modification of the activity of the complement system. We also probed the four complement genes located within the MHC region (i.e. *C2*, *C4A*, *C4B* and *CFB*) via schizophrenia PRS restricted to SNPs within each of them. We found strong significant associations with axonal density in all of them but *C4A*. Admittedly, and likely due to both local and long-range linkage disequilibrium within the region, the PRS calculated for these four genes were highly correlated, which could have confounded our results. In our data, the *C2* PRS was the best predictor of axonal density for all tracts, but no firm conclusions regarding the causal SNP for these associations can be drawn from our results. It is also important to highlight that previous evidence for *C4A* has centred around differences in expression due to structural variations in *C4* (copy number and insertion of an endogenous retrovirus [HERV]) [8], whereas our study focused on the potential effects of carrying a single risk SNPs for schizophrenia within this gene, regardless of its structural form. It is noteworthy, that a previous study also using complement PRS (excluding C4 SNPs from its calculation) found hippocampal volume to be associated with this score, but not with C4A predicted expression [29]; although structural variation within *C4A* has shown to be associated with changes in cortical surface area and thickness [24]. No previous study, though, had included white matter microstructure measures.

We also report an association between intergenic PRS for schizophrenia and axonal density, that we showed to be independent from genic and complement PRS. This intergenic association appeared stronger for chromosome 6 when divided by autosome, and explained by variants within the MHC region, as it was the case for complement PRS. However, in this case there was a significant amount of variance of axonal density also explained by SNPs outside this region. We report the intergenic PRS for chromosomes 4 and 19 to also associate to axonal density in different tracts (more strongly for chromosome 19), suggesting a potentially relevant role of intergenic regulatory factors. Future research should aim to replicate this finding and further investigate intergenic regions, which contain the majority of GWAS loci discovered in complex traits, to shed light into their potential influence on axonal density through regulatory mechanisms.

There are limitations to the interpretation of our results. Due to the complex linkage disequilibrium structure in the MHC region, where our main results are found, we cannot identify (i.e. “fine-map”) putative causal SNPs within this region driving the association with axonal density. Therefore, we cannot be conclusive on the role of complement genes included in this region or even prioritise specific genes. This is a common problem for results based on the MHC region in genetic association studies, which likely requires specialised sequencing protocols and bioinformatic analyses [30], and thus is not one that we can resolve here. Due to the nature of our study, we cannot link the reduction in axonal density associated with schizophrenia risk to over-pruning processes, and these could be explained by many other biological processes also happening early in development (e.g. neural proliferation and migration) or later in life (neural degeneration). Likewise, reduced axonal density in the context of NODDI could be indicative of different subjacent neurobiological alterations (e.g. thinner axons or lower myelination) that we cannot disentangle here. Future research using large samples with longitudinal data, targeting younger population and/or applying different imaging techniques that can offer deeper insights into the microstructure of white matter tracts (e.g., g-ratio [31]) and reproducibility of the findings reported here are needed. Previous research has shown widespread alterations in white matter tracts in participants with schizophrenia [13], in order to reduce the number of statistical tests and based on the dysconnectivity theory for schizophrenia, we have only included here association tracts innervating the frontal cortex; future research should also offer a wider picture of potential associations between complement PRS and white matter microstructure across the brain. Tentatively, though, we have analysed 2 further association tracts that do not innervate frontal cortex: the inferior longitudinal fasciculus and the posterior/parahippocampal cinculum. As can be seen in supplemental table 12, axonal density in the inferior longitudinal fasciculus shows very similar associations with genic, complement and intergenic schizophrenia PRS to the other tracts analysed here; however, axonal density in the posterior/parahippocampal cingulum does not seem to show any strong associations with any of these 3 schizophrenia PRS, suggesting that these associations do not generalise across all white matter tracts in the brain. Finally, due to the recruitment bias in UK Biobank [32] and the methods applied, our sample only included healthy white Europeans between 45 and 81 years of age, limiting our ability to extrapolate our results to the general population.

In conclusion, the results of this study show a significant association between common risk variants for schizophrenia contained within the MHC region in chromosome 6 and reduced axonal density in association white matter tracts innervating the frontal cortex, that could provide with a neuroanatomical basis for the differences in functional connectivity previously reported in the literature for patients [23] and individuals at high risk [33, 34]. Moreover, our results point towards a potential role of complement genes within this region in affecting axonal density, suggesting other potential mechanisms than risk variants within *C4A*, although further research is needed in this regard. Finally, potential regulatory intergenic signals within and outside the MHC region also showed to significantly associate with axonal density in the white matter tracts examined.

### Supplementary information

Supplemental Methods

Supplemental tables

## Data Availability

Data used in this study is publicly available and can be obtained via UK Biobank upon application from https://www.ukbiobank.ac.uk/enable-your-research.

## References

[1] Sullivan PF, Kendler KS, Neale MC (2003). Schizophrenia as a complex trait: evidence from a meta-analysis of twin studies. Arch Gen Psychiatry.

[2] Trubetskoy V, Pardiñas AF, Qi T, Panagiotaropoulou G, Awasthi S, Bigdeli TB (2022). Mapping genomic loci implicates genes and synaptic biology in schizophrenia. Nature.

[3] Choi SW, Mak TS, O’Reilly PF (2020). Tutorial: a guide to performing polygenic risk score analyses. Nat Protoc.

[4] Stauffer EM, Bethlehem RAI, Warrier V, Murray GK, Romero-Garcia R, Seidlitz J (2021). Grey and white matter microstructure is associated with polygenic risk for schizophrenia. Mol Psychiatry.

[5] Grama S, Willcocks I, Hubert JJ, Pardiñas AF, Legge SE, Bracher-Smith M (2020). Polygenic risk for schizophrenia and subcortical brain anatomy in the UK Biobank cohort. Transl Psychiatry.

[6] Coulthard LG, Hawksworth OA, Woodruff TM (2018). Complement: The Emerging Architect of the Developing Brain. Trends Neurosci.

[7] Schafer DP, Lehrman EK, Kautzman AG, Koyama R, Mardinly AR, Yamasaki R (2012). Microglia sculpt postnatal neural circuits in an activity and complement-dependent manner. Neuron.

[8] Sekar A, Bialas AR, de Rivera H, Davis A, Hammond TR, Kamitaki N (2016). Schizophrenia risk from complex variation of complement component 4. Nature.

[9] Woo JJ, Pouget JG, Zai CC, Kennedy JL (2020). The complement system in schizophrenia: where are we now and what’s next?. Mol Psychiatry.

[10] Sellgren CM, Gracias J, Watmuff B, Biag JD, Thanos JM, Whittredge PB (2019). Increased synapse elimination by microglia in schizophrenia patient-derived models of synaptic pruning. Nat Neurosci.

[11] Schmitt A, Hasan A, Gruber O, Falkai P (2011). Schizophrenia as a disorder of disconnectivity. Eur Arch Psychiatry Clin Neurosci.

[12] Friston KJ, Frith CD (1995). Schizophrenia: a disconnection syndrome?. Clin Neurosci.

[13] Kelly S, Jahanshad N, Zalesky A, Kochunov P, Agartz I, Alloza C (2018). Widespread white matter microstructural differences in schizophrenia across 4322 individuals: results from the ENIGMA Schizophrenia DTI Working Group. Mol Psychiatry.

[14] Zhang H, Schneider T, Wheeler-Kingshott CA, Alexander DC (2012). NODDI: practical in vivo neurite orientation dispersion and density imaging of the human brain. Neuroimage.

[15] Bycroft C, Freeman C, Petkova D, Band G, Elliott LT, Sharp K (2018). The UK Biobank resource with deep phenotyping and genomic data. Nature.

[16] Frankish A, Diekhans M, Ferreira AM, Johnson R, Jungreis I, Loveland J (2019). GENCODE reference annotation for the human and mouse genomes. Nucleic Acids Res.

[17] Carpanini SM, Harwood JC, Baker E, Torvell M, The Gerad C, Sims R, et al. The Impact of Complement Genes on the Risk of Late-Onset Alzheimer’s Disease. Genes (Basel) 2021;12:443. 10.3390/genes12030443 PMC8003605 33804666

[18] Werme J, van der Sluis S, Posthuma D, de Leeuw CA (2022). An integrated framework for local genetic correlation analysis. Nat Genet.

[19] Franke B, Stein JL, Ripke S, Anttila V, Hibar DP, van Hulzen KJE (2016). Genetic influences on schizophrenia and subcortical brain volumes: large-scale proof of concept. Nat Neurosci.

[20] Reus LM, Shen X, Gibson J, Wigmore E, Ligthart L, Adams MJ (2017). Association of polygenic risk for major psychiatric illness with subcortical volumes and white matter integrity in UK Biobank. Sci Rep.

[21] Alnæs D, Kaufmann T, van der Meer D, Córdova-Palomera A, Rokicki J, Moberget T (2019). Brain Heterogeneity in Schizophrenia and Its Association With Polygenic Risk. JAMA Psychiatry.

[22] Caseras X, Tansey KE, Foley S, Linden D (2015). Association between genetic risk scoring for schizophrenia and bipolar disorder with regional subcortical volumes. Transl Psychiatry.

[23] Váša F, Bullmore ET, Patel AX (2018). Probabilistic thresholding of functional connectomes: Application to schizophrenia. Neuroimage.

[24] O'Connell KS, Sønderby IE, Frei O, van der Meer D, Athanasiu L, Smeland OB (2021). Association between complement component 4A expression, cognitive performance and brain imaging measures in UK Biobank. Psychol Med.

[25] Bralten J, Franke B, Arias-Vásquez A, Heister A, Brunner HG, Fernández G (2011). CR1 genotype is associated with entorhinal cortex volume in young healthy adults. Neurobiol Aging.

[26] Allswede DM, Zheutlin AB, Chung Y, Anderson K, Hultman CM, Ingvar M (2018). Complement Gene Expression Correlates with Superior Frontal Cortical Thickness in Humans. Neuropsychopharmacology.

[27] Sun BB, Maranville JC, Peters JE, Stacey D, Staley JR, Blackshaw J (2018). Genomic atlas of the human plasma proteome. Nature.

[28] Thareja G, Belkadi A, Arnold M, Albagha OME, Graumann J, Schmidt F (2022). Differences and commonalities in the genetic architecture of protein quantitative trait loci in European and Arab populations. Hum Mol Genet.

[29] Holland JF, Cosgrove D, Whitton L, Harold D, Corvin A, Gill M (2020). Effects of complement gene-set polygenic risk score on brain volume and cortical measures in patients with psychotic disorders and healthy controls. Am J Med Genet B Neuropsychiatr Genet.

[30] Norman PJ, Norberg SJ, Guethlein LA, Nemat-Gorgani N, Royce T, Wroblewski EE (2017). Sequences of 95 human MHC haplotypes reveal extreme coding variation in genes other than highly polymorphic HLA class I and II. Genome Res.

[31] Jung W, Lee J, Shin HG, Nam Y, Zhang H, Oh SH (2018). Whole brain g-ratio mapping using myelin water imaging (MWI) and neurite orientation dispersion and density imaging (NODDI). Neuroimage.

[32] Fry A, Littlejohns TJ, Sudlow C, Doherty N, Adamska L, Sprosen T (2017). Comparison of Sociodemographic and Health-Related Characteristics of UK Biobank Participants With Those of the General Population. Am J Epidemiol.

[33] Morey RA, Inan S, Mitchell TV, Perkins DO, Lieberman JA, Belger A (2005). Imaging frontostriatal function in ultra-high-risk, early, and chronic schizophrenia during executive processing. Arch Gen Psychiatry.

[34] Bulbul O, Kurt E, Ulasoglu-Yildiz C, Demiralp T, Ucok A (2022). Altered Resting State Functional Connectivity and Its Correlation with Cognitive Functions at Ultra High Risk for Psychosis. Psychiatry Res Neuroimaging.

