# Supplementary material for: Common risk alleles for schizophrenia within the major histocompatibility complex predict white matter microstructure
Source: Transl Psychiatry. 2024 Apr 22;14:194. doi: 10.1038/s41398-024-02910-2 (PMC11035599; doi:10.1038/s41398-024-02910-2)
Supplement: Supplementary file 1 — Supplemental Methods [file 41398_2024_2910_MOESM1_ESM.docx]

**Supplemental methods**

A total of 36,846 individuals had non-missing data on all 4 bilateral neurite density measures and were included in the downstream analyses. Prior to GWAS, each measure was standardized and converted to a z-score. The distribution of each measure was approximately normal, with a mean of 0 and a standard deviation of 1 (Figure 1).


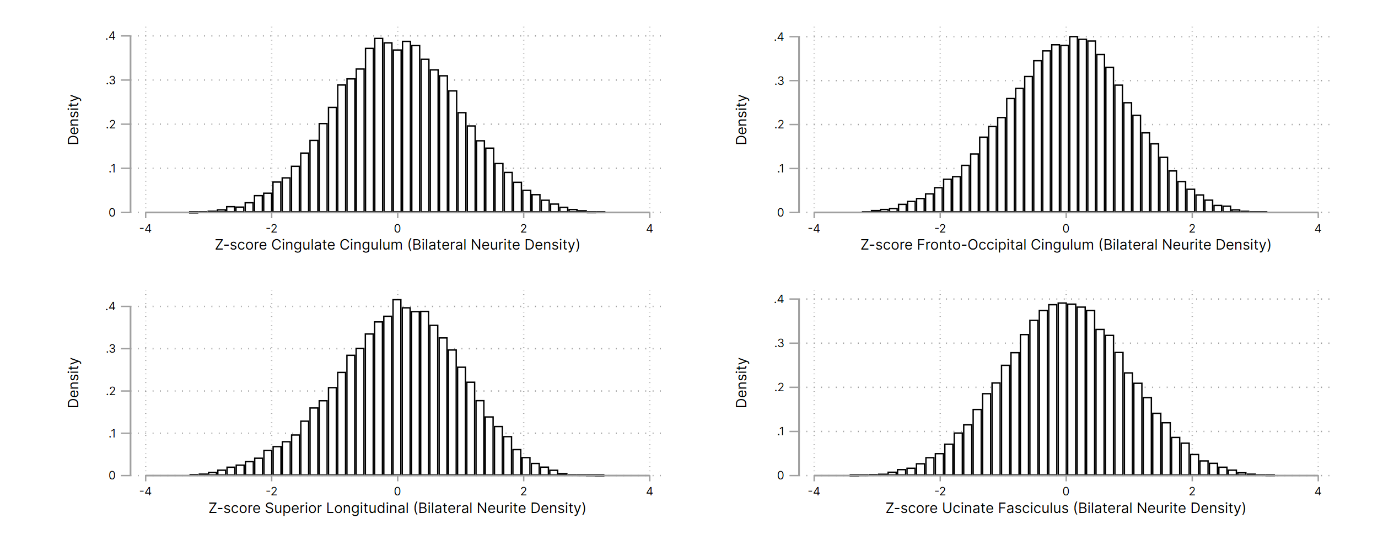


Figure 1: Distribution of bilateral neurite density measures included in GWAS.

We limited genotype quality control to the subset of individuals who were included in the brain imaging analyses. Briefly, we limited data to a minor allele count of 5, harmonization to a common build (hg19), removed individuals with excessive relatedness, with missingness of greater than 2% and excessive homozygosity or heterozygosity compared to the sample mean. Phase 1 and Phase 2 included 20665 and 25650 individuals respectively. Genetic variation was limited to bi-allelic markers, markers were excluded if there were sample Hardy-Weinberg deviation of p < 10^-20^, allele frequency deviated from ancestry-predicted value (as defined in ALFA), or if there was missingness of greater than 2%. Phase 1 and Phase 2 included 5696478 and 6068166 variants respectively.

Each dataset was further restricted to individuals whose genotypes were “genetically like” those of the 1000 genotypes GBR reference dataset as defined using ancestry principal components, specifically within 4 standard deviations of the reference mean over the first 5 principal components (Figure 2). The processed genotype data were merged into a single dataset and further limited to an unrelated subset using the relationship-based pruning routine (--king-cutoff) in PLINK (threshold = 0.0442). A total of 5612413 variants in a dataset of 32692 GBR-like unrelated individuals with complete MRI data were retained for downstream analysis.

A


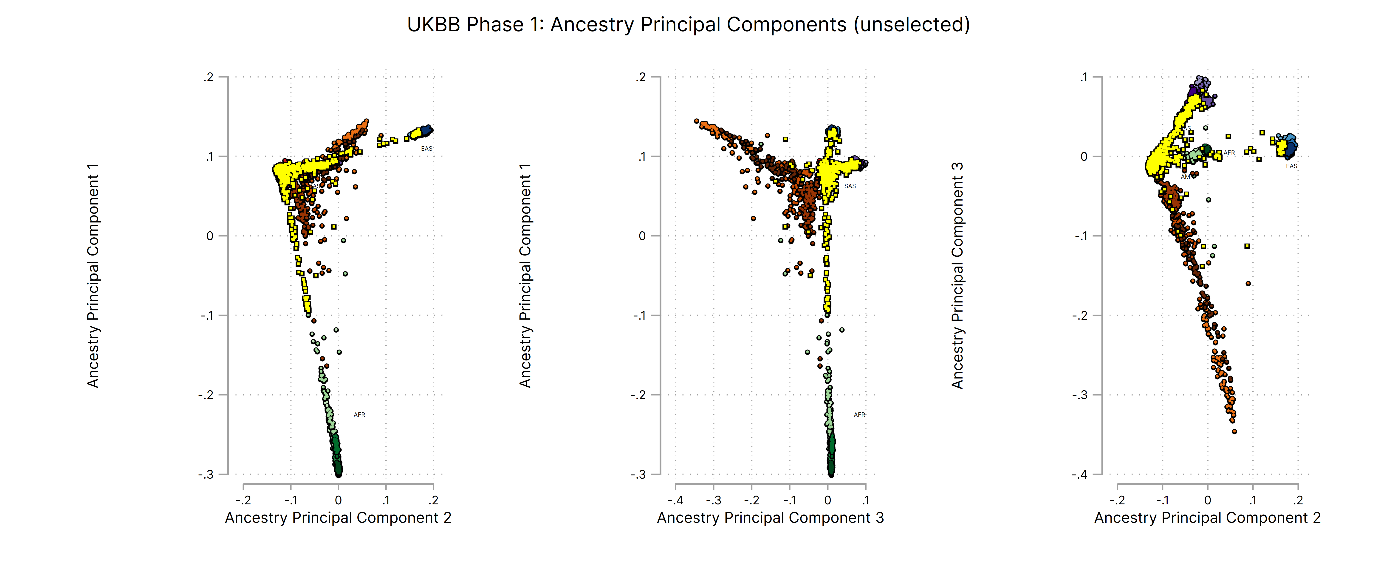


B


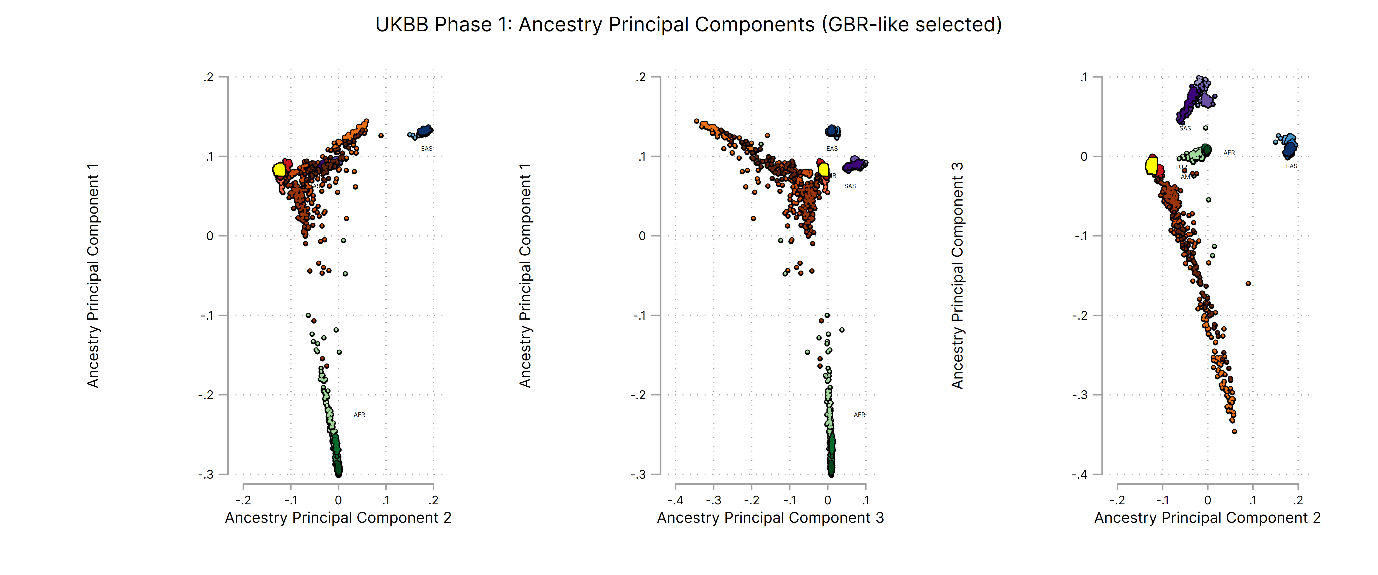


Figure 2: Ancestry principal components for UKBB Brain Imaging Phase 1 dataset. UK Biobank individuals shown in yellow. Reference ancestries from 1000 genomes project; Blue = East Asia, Green = Africa, Red = Europe, Orange = Americas, and Purple = South Asia. Panel A shows the unselected individuals across the first 3 PCs. Panel B shows the data subset limited to GBR-like individuals.

GWAS was performed for each bilateral neurite density using generalized-linear model in PLINK, adjusted for sex, age at scan (in years), scan centre, and 10 ancestry principal component. Summary Manhattan Plots for each analysis are described in Figure 3.


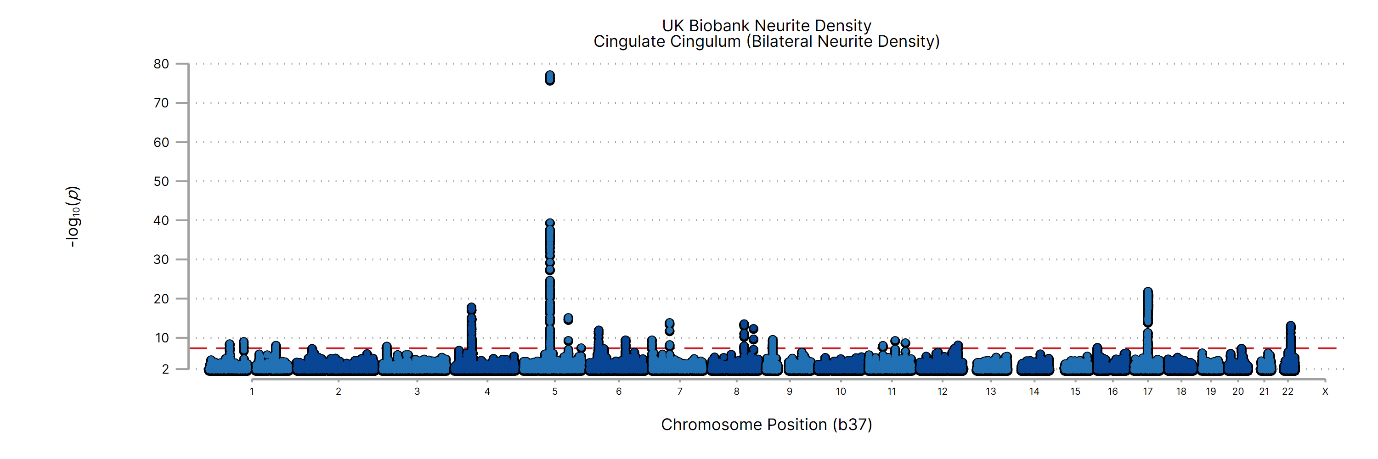

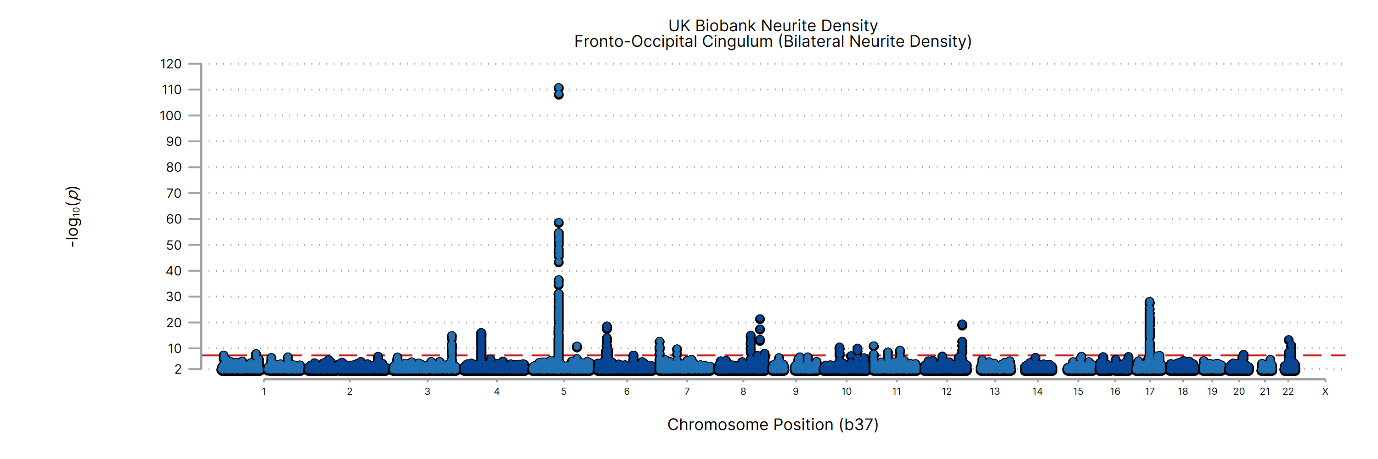

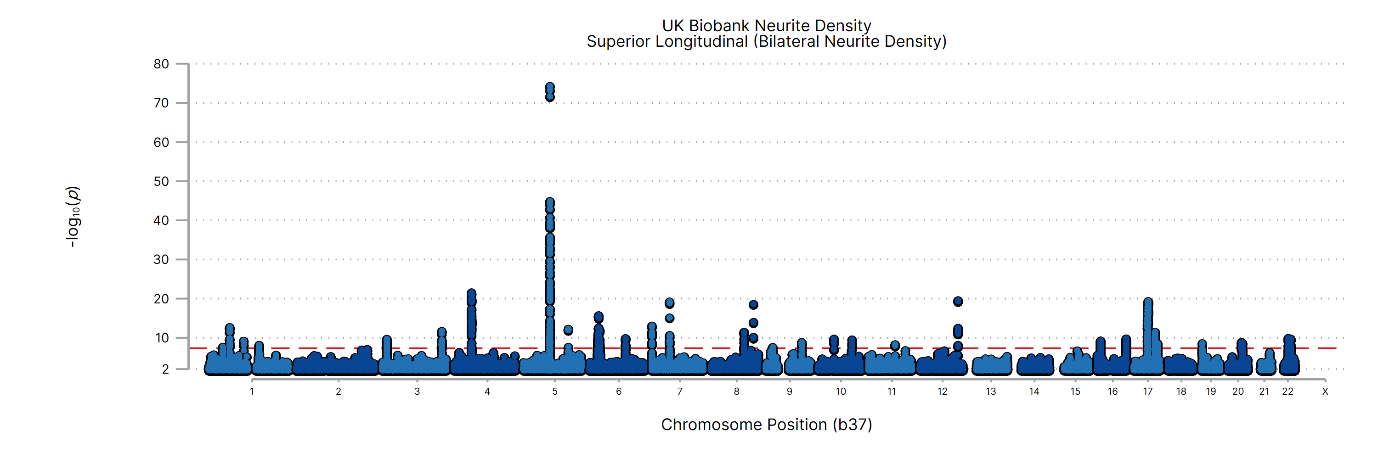

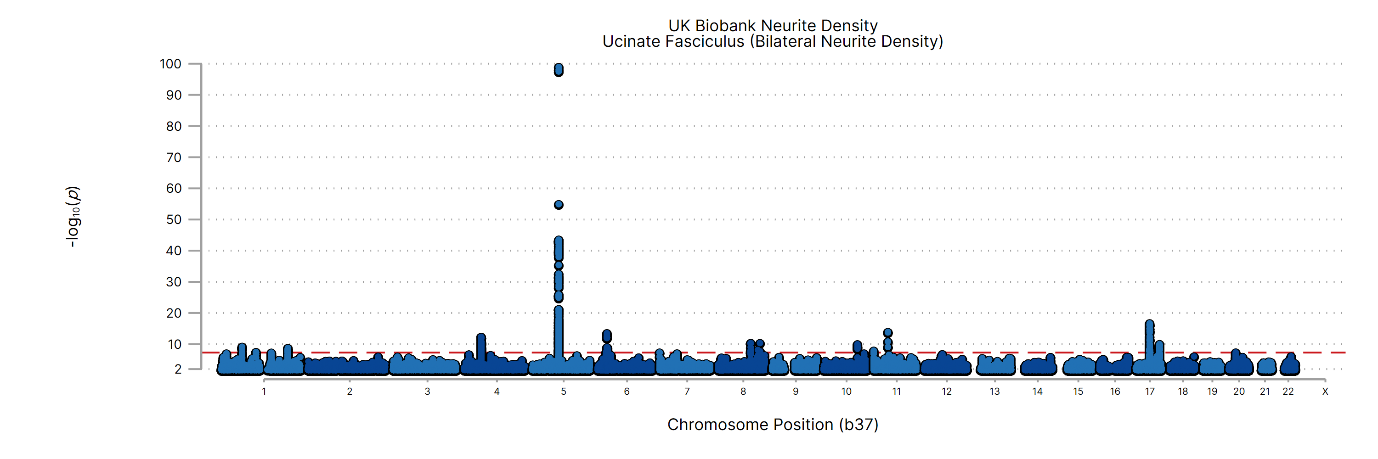


Figure 3: Manhattan Plots for GWAS on bilateral neurite density in combined UK Biobank MGI dataset.

The SNP heritability for each summary dataset was estimated using LDSC. SNP heritability was calculated on a subset of SNPs included in the w_hm3 SNP panel. A summary of the observed heritability for each of the 4 traits is described in Table 1.

Table 1: Summary of SNP heritability for the bilateral neurite density measures. Estimates calculated using ldsc.py limited to the w_hm3 SNP panel.

| Phenotype | Heritability | Standard Error | Intercept |
| --- | --- | --- | --- |
| Cingulate Cingulum | 0.3615 | 0.035 | 1.01 |
| Fronto-Occipital Cingulum | 0.3590 | 0.037 | 1.02 |
| Superior Longitudinal | 0.3971 | 0.039 | 1.02 |
| Ucinate Fasciculus | 0.2269 | 0.031 | 1.02 |

Similar GWAS, limited to unilateral measures have previously been performed as part Big40 project. Summary GWAS statistics for these analyses is available at <https://open.win.ox.ac.uk/ukbiobank/big40/>. Specifically, we downloaded and harmonized the GWAS summary data for weighted mean intra-cellular volume fraction for the left and right hemisphere tracts (Table 2). We compared the genetic correlation between the bilateral measures and previously reported unilateral neurite density measures from the Big40 study (Table 3).

Table 2: Description of GWAS summary data downloaded from Big40. Phenotype refers to the Big40 study identifier. Additional SNP heritability for the unilateral neurite density measures is included for comparison. Estimates calculated locally using ldsc.py limited to the w_hm3 SNP panel.

| Phenotype | Description of Measure | Heritability | Standard Error | Intercept |
| --- | --- | --- | --- | --- |
| 1954 | Cingulate Cingulum (Left) | 0.3239 | 0.033 | 1.02 |
| 1955 | Cingulate Cingulum (Right) | 0.3262 | 0.032 | 1.02 |
| 1962 | Fronto-Occipital Cingulum (Left) | 0.3630 | 0.040 | 1.02 |
| 1963 | Fronto-Occipital Cingulum (Right) | 0.3689 | 0.040 | 1.02 |
| 1971 | Superior Longitudinal (Left) | 0.3969 | 0.041 | 1.02 |
| 1972 | Superior Longitudinal (Right) | 0.4021 | 0.041 | 1.02 |
| 1975 | Ucinate Fasciculus (Left) | 0.3429 | 0.031 | 1.01 |
| 1976 | Ucinate Fasciculus (Right) | 0.3637 | 0.033 | 1.01 |

Table 3: Genetic correlation between unilateral and bilateral neurite density measures. Note 1: the ldsc.py regression is not a bounded estimator (i.e. the correlations are not bounded between -1 and 1). Estimates can exceed these bounds due to sampling variation.

| Phenotype | Genetic Correlation (rg) with Standard Error | | |
| --- | --- | --- | --- |
|  | Left vs. Right | Bilateral vs. Left | Bilateral vs. Right |
| Cingulate Cingulum | 0.966 (0.010) | 1.006 (0.009) | 0.997 (0.010) |
| Fronto-Occipital Cingulum | 0.998 (0.002) | 1.012 (0.008) | 1.008 (0.007) |
| Superior Longitudinal | 0.997 (0.002) | 1.007 (0.007) | 1.011 (0.007) |
| Ucinate Fasciculus | 0.973 (0.006) | 1.002 (0.010) | 0.997 (0.009) |

Summary statistics from these GWAS analyses are available on request to the authors.
