# Supplementary material for: Common risk alleles for schizophrenia within the major histocompatibility complex predict white matter microstructure
Source: Transl Psychiatry. 2024 Apr 22;14:194. doi: 10.1038/s41398-024-02910-2 (PMC11035599; doi:10.1038/s41398-024-02910-2)
Supplement: Supplementary file 2 — Supplemental tables [file 41398_2024_2910_MOESM2_ESM.docx]

Supplemental table 1. List of genes included within the Complement gene-set

| Chromosome | BP-start | BP-end | Gene | Entrez_ID |
| --- | --- | --- | --- | --- |
| 1  1  1  1  1  1  1  1  1  1  1  1  1  1  1  1  1  1  1  1  2  3  4  5  5  5  6  6  6  6  8  8  9  9  9  9  10  10  11  11  12  12  12  12  12  16  16  17  17  17  19  19  19  19  20 | 27695603  196743925  57320479  57394883  207818458  196788898  22970123  22979255  22962999  11086580  196621008  207669492  196788887  207494853  207262187  207277607  196819371  207925402  207627575  196946667  119913819  186935942  110661852  40909354  39284364  41142336  31895475  31982539  31949801  31865562  2792875  27454434  137772654  139839698  137801431  123714616  16555742  54525140  57364860  33719807  49726200  8210898  7096351  7187513  7242183  31366455  31271311  5336097  43037061  26691290  859453  47835404  6677715  47793280  23059986 | 27701315  196763203  57383894  57431813  207911761  196928356  22974603  22988031  22966101  11107290  196716634  207813992  196801319  207534311  207273338  207318317  196888102  207968858  207663240  196978804  119916465  187009810  110723335  40983041  39424970  41261540  31919861  32003195  31970458  31913449  4852494  27472548  137779924  139841426  137809809  123812554  16564004  54531460  57382326  33757991  49730971  8219067  7178336  7245203  7261869  31394318  31344213  5352150  43045439  26700110  863453  47846606  6730573  47825323  23066977 | FCN3  CFHR3  C8A  C8B  CR1L  CFHR2  C1QC  C1QB  C1QA  MASP2  CFH  CR1  CFHR1  CD55  C4BPB  C4BPA  CFHR4  CD46  CR2  CFHR5  C1QL2  MASP1  CFI  C7  C9  C6  CFB  C4B  C4A  C2  CSMD1  CLU  FCN2  C8G  FCN1  C5  C1QL3  MBL2  SERPING1  CD59  C1QL4  C3AR1  C1S  C1R  C1RL  ITGAX  ITGAM  C1QBP  C1QL1  VTN  CFD  C5AR2  C3  C5AR1  CD93 | 8547  10878  731  732  1379  3080  714  713  712  10747  3075  1378  3078  1604  725  722  10877  4179  1380  81494  165257  5648  3426  730  735  729  629  721  720  717  64478  1191  2220  733  2219  727  389941  4153  710  966  338761  719  716  715  51279  3687  3684  708  10882  7448  1675  27202  718  728  22918 |

BP-start: start break point; BP-end: ending break point; Entrez_ID: NCBI gene identifier

Supplemental table 2. SNP count for each PS (Genic, Complement and Intergenic) at each p threshold used

|  | Schizophrenia | | |
| --- | --- | --- | --- |
|  | Genic | Intergenic | Complement |
| p-threshold |  |  |  |
| 0.1  0.01  0.001  0.0001  0.00001 | 66,263  16,451  4,782  1,782  826 | 47,010  11,628  3,415  1,250  570 | 486  126  38  18  11 |
|  | Schizophrenia minus MHC | | |
|  | Genic | Intergenic | Complement |
| p-threshold |  |  |  |
| 0.1  0.01  0.001  0.0001  0.00001 | 65,955  16,269  4,687  1,717  783 | 46,837  11,541  3,363  1,217  550 | 472  120  34  15  9 |

Supplemental table 3. Genic, Complement and Intergenic schizophrenia polygenic risk scores calculated at 5 progressive p thresholds, and their association (beta[p]) with axonal density in association tracts.

|  | CG | iFO | sL | Unc |
| --- | --- | --- | --- | --- |
| Genic PRS  0.1  0.01  0.001  0.0001  0.00001 | -.008 (.15)  -.009 (.09)  -.013 (.01)  -.017 (.001)  -.014 (.008) | -.011 (.04)  -.013 (.01)  -.013 (.01)  -.015(.006)  -.015 (.005) | **-.018 (.0008)**  -.016 (.003)  -.016 (.003)  -.017 (.001)  -.016 (.004) | -.002 (.76)  -.007 (.20)  -.008 (.12)  -.009 (.10)  -.008 (.15) |
| Complement PRS  0.1  0.01  0.001  0.0001  0.00001 | .002 (.78)  -.002 (.77)  -.017 (.002)  **-.020 (.0001)**  **-.022 (4x10^-5^)** | -.010 (.07)  -.011 (.03)  **-.022 (4x10^-5^)**  **-.024 (6x10^-7^)**  **-.028 (1x10^-7^)** | -.011 (.04)  -.010 (.07)  **-.022 (7x10^-5^)**  **-.026 (2x10^-6^)**  **-.028 (4x10^-7^)** | -.005 (.36)  -.005 (.37)  -.016 (.002)  **-.022 (4x10^-5^)**  **-.026 (1x10^-6^)** |
| Intergenic PRS  0.1  0.01  0.001  0.0001  0.00001 | -.011 (.04)  -.015 (.004)  -.017 (.001)  **-.025 (4x10^-6^)**  **-.023 (2x10^-5^)** | -.009(.08)  **-.020 (.0001)**  **-.022 (4x10^-5^)**  **-.026 (1x10^-6^)**  **-.027 (3x10^-7^)** | -.011 (.05)  **-.022 (5x10^-5^)**  **-.020 (.0002)**  **-.025 (4x10^-6^)**  **-.026 (2x10^-6^)** | -.005 (.32)  -.012 (.02)  -.014 (.01)  **-.020 (.0001)**  **-.022 (4x10^-5^)** |

CG: cingulum-cingulate gyrus part, iFO: inferior fronto-occipital fasciculus, sL: superior longitudinal fasciculus, Unc: uncinate fasciculus.

Bonferroni corrected (60 tests) significant results are highlighted in bold.

Supplemental table 4. Complement, intergenic and genic schizophrenia polygenic risk scores calculated at 5 progressive p thresholds, and their association (beta[p]) with orientation dispersion when entered simultaneously in the same regression model.

|  | CG | iFO | sL | Unc |
| --- | --- | --- | --- | --- |
| p-threshold 0.1  Complement PRS  Intergenic PRS  Genic PRS  p-threshold 0.01  Complement PRS  Intergenic PRS  Genic PRS  p-threshold 0.001  Complement PRS  Intergenic PRS  Genic PRS  p-threshold 0.0001  Complement PRS  Intergenic PRS  Genic PRS  p-threshold 0.00001  Complement PRS  Intergenic PRS  Genic PRS | -.002 (.68)  -.009 (.10)  -.005 (.37)  ~ .00 (.92)  -.013 (.01)  -.005 (.32)  -.015 (.005)  -.014 (.01)  -.007 (.19)  -.017 (.002)  -.020 (.0004)  -.010 (.07)  -.018 (.0006)  -.018 (.0009)  -.007 (.22) | -.012 (.02)  -.007 (.23)  -.008 (.14)  -.010 (.07)  -.018 (.001)  -.008 (.16)  -.020 (.0002)  -.019 (.0007)  -.006 (.29)  -.021 (.00009)  -.022 (.00009)  -.006 (.29)  -.024 (.000009)  -.022 (.00005)  -.006 (.30) | -.009 (.09)  -.005 (.36)  -.016 (.004)  -.008 (.14)  -.018 (.001)  -.011 (.05)  -.019 (.0003)  -.015 (.006)  -.010 (.08)  -.023 (.00004)  -.020 (.0003)  -.009 (.11)  -.024 (.00001)  -.021 (.0002)  -.007 (.21) | -.008 (.15)  -.003 (.54)  ~ .00 (.96)  -.004 (.46)  -.010 (.05)  -.004 (.50)  -.015 (.004)  -.011 (.04)  -.004 (.52)  -.020 (.0002)  -.018 (.0009)  -.001 (.85)  -.023 (.00001)  -.019 (.0005)  .001 (.13) |

CG: cingulum-cingulate gyrus part, iFO: inferior fronto-occipital fasciculus, sL: superior longitudinal fasciculus,

Unc: uncinate fasciculus; Pt: p threshold.

Supplemental table 5. Top five signals from the Schizophrenia GWAS (PGC3) included in the Complement and Intergenic PRS

|  | Chromosome | SNP | A1 | A2 | Beta | SE Beta | p |
| --- | --- | --- | --- | --- | --- | --- | --- |
| Complement | 6  8  6  8  8 | rs497309  rs73229090  rs2736428  rs10503253  rs7464891 | A  C  C  C  G | C  A  T  A  A | 0.1557  0.1021  -0.0564  -0.0594  0.0446 | 0.014  0.014  0.008  0.009  0.008 | 1.17*10^-26^  6.19*10^-13^  1.22*10^-11^  7.73*10^-11^  2.89*10^-07^ |
| Intergenic | 6  6  6  6  6 | rs13195636  rs13194504  rs3115631  rs34107459  rs3132090 | A  G  T  T  G | C  A  A  C  A | 0.2088  0.2062  0.1780  0.1885  0.1532 | 0.016  0.016  0.015  0.016  0.014 | 4.51*10^-39^  9.34*10^-37^  5.26*10^-29^  6.09*10^-29^  1.44*10^-27^ |

All the above signals from Chromosome 6 located within the defined MCH region (25-35Mb)

Supplemental table 6. Association (beta[p]) of two quasi-independent risk SNP for schizophrenia within the MHC region with axonal density in association tracts when added simultaneously into a regression model.

|  | CG | iFO | sL | Unc |
| --- | --- | --- | --- | --- |
| rs13195636  rs8192589 | -.023 (.0001)  -.008 (.19) | -.021 (.0005)  -.021 (.0005) | -.017 (.006)  -.021 (.0005) | -.021 (.0004)  -.014 (.019) |

Risk variant rs13195636 (A), rs8192589 (G)

CG: cingulum-cingulate gyrus part, iFO: inferior fronto-occipital fasciculus, sL: superior longitudinal fasciculus, Unc: uncinate fasciculus.

Supplemental table 7. Linkage Disequilibrium (r2) between SNPs included in the polygenic score calculations for C4A, C4B, C2 and CFB.

| Gene  (location) | SNP | rs497309 | rs7887 | rs17421133 | rs1150757 | rs7766862 | rs61745355 | rs1270942 |
| --- | --- | --- | --- | --- | --- | --- | --- | --- |
| C2  (31862785-31955672) | rs497309  rs7887 | ----  .072 | ---- |  |  |  |  |  |
| C4A  (31947024-32012681) | rs17421133 | .061 | .061 | ---- |  |  |  |  |
| C4B  (31979795-32045418 | rs1150757  rs7766862  rs61745355 | .975  .061  .003 | .071  .607  .010 | .063  .982  .009 | ----  .063  .003 | ----  .009 | ---- |  |
| CFB  (31910650-31962084) | rs1270942  rs2280774 | .993  .065 | .072  .723 | .061  .825 | .982  .064 | .061  .817 | .003  .009 | ----  .065 |

The locations of each gene include the 35kb upstream and 10kb downstream accounted.

Supplemental table 8. Association (beta[p]) of schizophrenia polygenic risk score for Complement genes located in the MHC region with axonal density when added simultaneously into a regression model.

|  | n SNPs | CG | iFO | sL | Unc |
| --- | --- | --- | --- | --- | --- |
| C2  C4A  C4B  CFB | 2  1  3  2 | **-.086 (.002)**  -.009 (.14)  -.022 (.17)  .080 (.013) | -.055 (.049)  .011 (.09)  -.018 (.27)  .034 (.29) | -.054 (.056)  .014 (.03)  -.028 (.09)  .044 (.18) | -.039 (.15)  .008 (.21)  -.012 (.45)  .022 (.48) |

n SNPs: number of SNPs included in the PRS after clumping.

CG: cingulum-cingulate gyrus part, iFO: inferior fronto-occipital fasciculus, sL: superior longitudinal fasciculus, Unc: uncinate fasciculus.

Bonferroni corrected (16 tests) significant results are highlighted in bold.

Supplemental table 9. Associations (beta[p]) between autosome intergenic and genic polygenic risk scores for schizophrenia calculated at p threshold <.0001 and axonal density in association tracts when added simultaneously into a regression model.

|  | CG | iFO | sL | Unc |
| --- | --- | --- | --- | --- |
| Chromosome 1  Intergenic PRS  Genic PRS | -.010 (.07)  -.018 (.002) | -.005 (.36)  -.015 (.008) | -.006 (.32)  -.018 (.002) | -.010 (.08)  -.018 (.002) |
| Chromosome 4  Intergenic PRS  Genic PRS | -.004 (.49)  -.013 (.01) | -.012 (.02)  -.008 (.14) | -.015 (.006)  -.009 (.12) | -.007 (.20)  -.006 (.28) |
| Chromosome 19  Intergenic PRS  Genic PRS | -.015 (.007)  ~ .00 (.97) | -.019 (.0004)  .001 (.78) | -.017 (.001)  -.001 (.82) | -.018 (.0008)  .002 (.67) |

CG: cingulum-cingulate gyrus part, iFO: inferior fronto-occipital fasciculus, sL: superior longitudinal fasciculus, Unc: uncinate fasciculus.

Supplemental table 10. Genic, Complement and Intergenic schizophrenia polygenic risk scores calculated at 5 progressive p thresholds, and their association (beta[p]) with orientation dispersion in association tracts.

|  | CG | iFO | sL | Unc |
| --- | --- | --- | --- | --- |
| Genic PS  0.1  0.01  0.001  0.0001  0.00001 | .006 (.29)  .009 (.10)  .015 (.008)  .014 (.01)  .013 (.02) | .011 (.04)  .008 (.14)  .010 (.07)  .005 (.32)  .004 (.47) | -.003 (.57)  -.004 (.51)  -.001 (.80)  -.004 (.42)  .005 (.31) | .011 (.04)  .015 (.008)  .011 (.05)  .010 (.07)  .016 (.004) |
| Complement PS  0.1  0.01  0.001  0.0001  0.00001 | .002 (.78)  -.009 (.09)  .005 (.38)  .004 (.48)  .005 (.33) | -.010 (.07)  -.003 (.64)  -.002 (.67)  -.004 (.49)  -.001 (.85) | -.011 (.04)  -.009 (.12)  -.004 (.50)  -.004 (.46)  -.005 (.39) | -.005 (.36)  <.001 (.98)  .002 (.75)  .001 (.87)  .001 (.90) |
| Intergenic PS  0.1  0.01  0.001  0.0001  0.00001 | .004 (.44)  .015 (.008)  .011 (.04)  .013 (.01)  .011 (.04) | -.003 (.64)  .004 (.50)  <.001 (.93)  .002 (.68)  <.001 (.99) | -.008 (.15)  -.011 (.04)  -.004 (.45)  -.005 (.38)  -.002 (.76) | .006 (.28)  <.001 (.98)  .001 (.92)  .010 (.07)  .007 (.21) |

CG: cingulum-cingulate gyrus part, iFO: inferior fronto-occipital fasciculus, sL: superior longitudinal fasciculus, Unc: uncinate fasciculus.

Supplemental table 11. Main results from the LAVA analyses including summary statistics from PGC3 GWAS for schizophrenia and UK Biobank GWASes for axonal density in each of the white matter tracts investigated. Only bivariate genetic correlations surviving a Bonferroni correction for all regions with significant local heritability across all axonal density phenotypes (1413 overall tests) are shown.

| **Locus** | **Chrom.** | **start** | **stop** | **n.SNPs** | **Tract** | **rho** | **rho.low** | **rho.high** | **r2** | **r2.lower** | **r2.upper** | **p-value** |
| --- | --- | --- | --- | --- | --- | --- | --- | --- | --- | --- | --- | --- |
| 768 | 4 | 184338585 | 185483262 | 1481 | CG | 0.567 | 0.310 | 0.869 | 0.322 | 0.096 | 0.756 | 3.05×10^-5^ |
| 768 | 4 | 184338585 | 185483262 | 1481 | Unc | 0.648 | 0.368 | 0.986 | 0.420 | 0.136 | 0.972 | 1.58×10^-5^ |
| 919 | 5 | 172285683 | 173606995 | 1453 | CG | 0.524 | 0.301 | 0.776 | 0.275 | 0.091 | 0.603 | 1.45×10^-5^ |
| 954 | 6 | 29529756 | 29833843 | 1172 | iFO | -0.767 | -1.000 | -0.524 | 0.588 | 0.274 | 1.000 | 1.39×10^-6^ |
| 956 | 6 | 30070718 | 30715006 | 1270 | iFO | -0.763 | -1.000 | -0.525 | 0.583 | 0.275 | 1.000 | 5.52×10^-7^ |
| 956 | 6 | 30070718 | 30715006 | 1270 | Unc | -0.725 | -1.000 | -0.488 | 0.525 | 0.238 | 1.000 | 8.72×10^-7^ |
| 957 | 6 | 30715007 | 31106493 | 1153 | sL | -0.685 | -1.000 | -0.408 | 0.470 | 0.167 | 1.000 | 2.84×10^-5^ |
| 958 | 6 | 31106494 | 31250556 | 823 | iFO | -1.000 | -1.000 | -0.792 | 1.000 | 0.628 | 1.000 | 2.64×10^-10^ |
| 958 | 6 | 31106494 | 31250556 | 823 | sL | -0.957 | -1.000 | -0.750 | 0.915 | 0.562 | 1.000 | 2.29×10^-8^ |
| 958 | 6 | 31106494 | 31250556 | 823 | Unc | -0.981 | -1.000 | -0.778 | 0.963 | 0.605 | 1.000 | 4.00×10^-8^ |
| 959 | 6 | 31250557 | 31320268 | 546 | iFO | -0.911 | -1.000 | -0.708 | 0.830 | 0.501 | 1.000 | 4.11×10^-8^ |
| 959 | 6 | 31250557 | 31320268 | 546 | sL | -0.860 | -1.000 | -0.626 | 0.740 | 0.392 | 1.000 | 1.21×10^-6^ |
| 959 | 6 | 31250557 | 31320268 | 546 | Unc | -0.794 | -1.000 | -0.561 | 0.630 | 0.315 | 1.000 | 1.07×10^-6^ |
| 960 | 6 | 31320269 | 31427209 | 754 | iFO | -0.804 | -1.000 | -0.553 | 0.646 | 0.306 | 1.000 | 3.33×10^-6^ |
| 960 | 6 | 31320269 | 31427209 | 754 | sL | -0.758 | -1.000 | -0.508 | 0.574 | 0.258 | 1.000 | 5.40×10^-6^ |
| 961 | 6 | 31427210 | 32208901 | 1120 | iFO | -0.524 | -0.766 | -0.294 | 0.274 | 0.087 | 0.587 | 1.97×10^-5^ |
| 962 | 6 | 32208902 | 32454577 | 1017 | iFO | -0.737 | -1.000 | -0.473 | 0.543 | 0.223 | 1.000 | 9.46×10^-6^ |
| 1557 | 10 | 77144962 | 78665481 | 1327 | iFO | 0.619 | 0.370 | 0.911 | 0.383 | 0.137 | 0.829 | 7.41×10^-6^ |
| 1557 | 10 | 77144962 | 78665481 | 1327 | sL | 0.646 | 0.366 | 1.000 | 0.417 | 0.134 | 1.000 | 2.72×10^-5^ |
| 2207 | 17 | 43460501 | 44865832 | 1212 | CG | 0.771 | 0.598 | 0.952 | 0.595 | 0.358 | 0.907 | 2.94×10^-11^ |
| 2207 | 17 | 43460501 | 44865832 | 1212 | sL | 0.605 | 0.405 | 0.816 | 0.366 | 0.164 | 0.665 | 4.61×10^-7^ |
| 2207 | 17 | 43460501 | 44865832 | 1212 | Unc | 0.681 | 0.442 | 0.935 | 0.464 | 0.195 | 0.874 | 2.63×10^-6^ |
| 2474 | 22 | 29457602 | 30962255 | 1495 | iFO | -0.462 | -0.671 | -0.266 | 0.214 | 0.071 | 0.450 | 1.90×10^-5^ |
| 2474 | 22 | 29457602 | 30962255 | 1495 | sL | -0.507 | -0.723 | -0.305 | 0.257 | 0.093 | 0.522 | 5.96×10^-6^ |

Supplemental table 12. Genic, Complement and Intergenic schizophrenia polygenic risk scores calculated at 5 progressive p thresholds, and their association (beta[p]) with axonal density in two complementary tracts.

|  | PG | iL |
| --- | --- | --- |
| Genic PRS  0.1  0.01  0.001  0.0001  0.00001 | -.006 (.29)  -.007 (.22)  -.002 (.73)  -.004 (.44)  -.006 (.29) | -.013 (.01)  -.015 (.006)  -.017 (.002)  -.019(.0003)  -.017 (.001) |
| Complement PRS  0.1  0.01  0.001  0.0001  0.00001 | -.006 (.29)  -.001 (.83)  -.014 (.01)  -.012 (.02)  -.011 (.04) | -.013 (.02)  -.009 (.08)  -.022 (5x10^-5^)  -.025 (3x10^-6^)  -.029 (1x10^-7^) |
| Intergenic PRS  0.1  0.01  0.001  0.0001  0.00001 | -.005 (.32)  -.008 (.17)  -.008 (.13)  -.012 (.03)  -.012 (.02) | -.009 (.10)  -.021 (8x10^-5^)  -.022 (6x10^-5^)  -.026 (2x10^-6^)  -.025 (5x10^-6^) |

PCG: posterior/parahippocampal cingulum, iL: inferior longitudinal fasciculus.
